# Supplementary material for: Regio- and conformational isomerization critical to design of efficient thermally-activated delayed fluorescence emitters
Source: Nat Commun. 2017 Apr 13;8:14987. doi: 10.1038/ncomms14987 (PMC5399286; doi:10.1038/ncomms14987)
Supplement: Supplementary Information — Supplementary Figures, Supplementary Tables, Supplementary Methods and Supplementary References [file ncomms14987-s1.pdf]

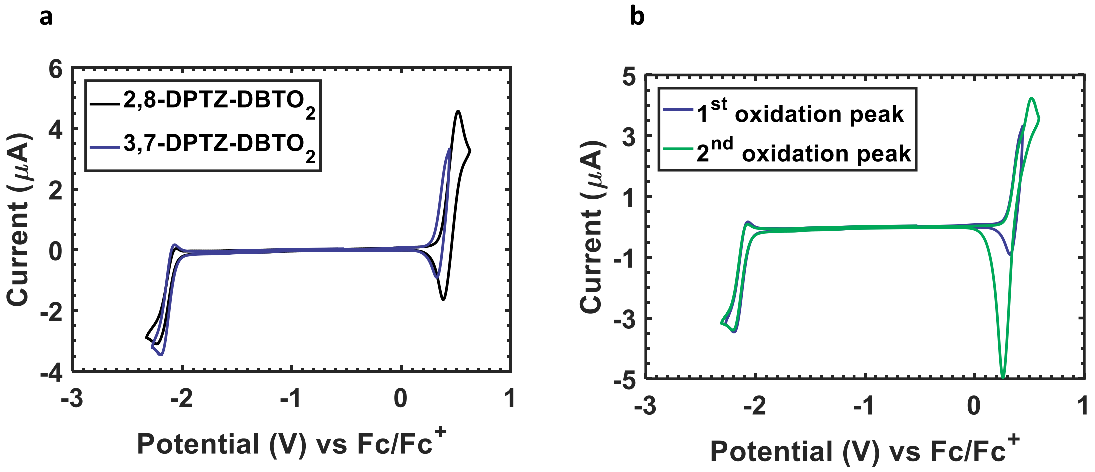

Supplementary Figure 1| Cyclic voltammetry of investigated compounds 2,8-DPTZ-DBTO$_2$ and 3,7-DPTZ-DBTO$_2$ at 1 mM concentration (**a**) in 0.1 M Bu_4NBF_4/DCM. (**b**) Comparison of CV curve up to first and second oxidation potential of 3,7-DPTZ-DBTO$_2$. Measurement conditions: scan rate 50 mV/s, Ag/AgCl – quasi-reference electrode, calibrated against a ferrocene/ferrocenium redox couple.

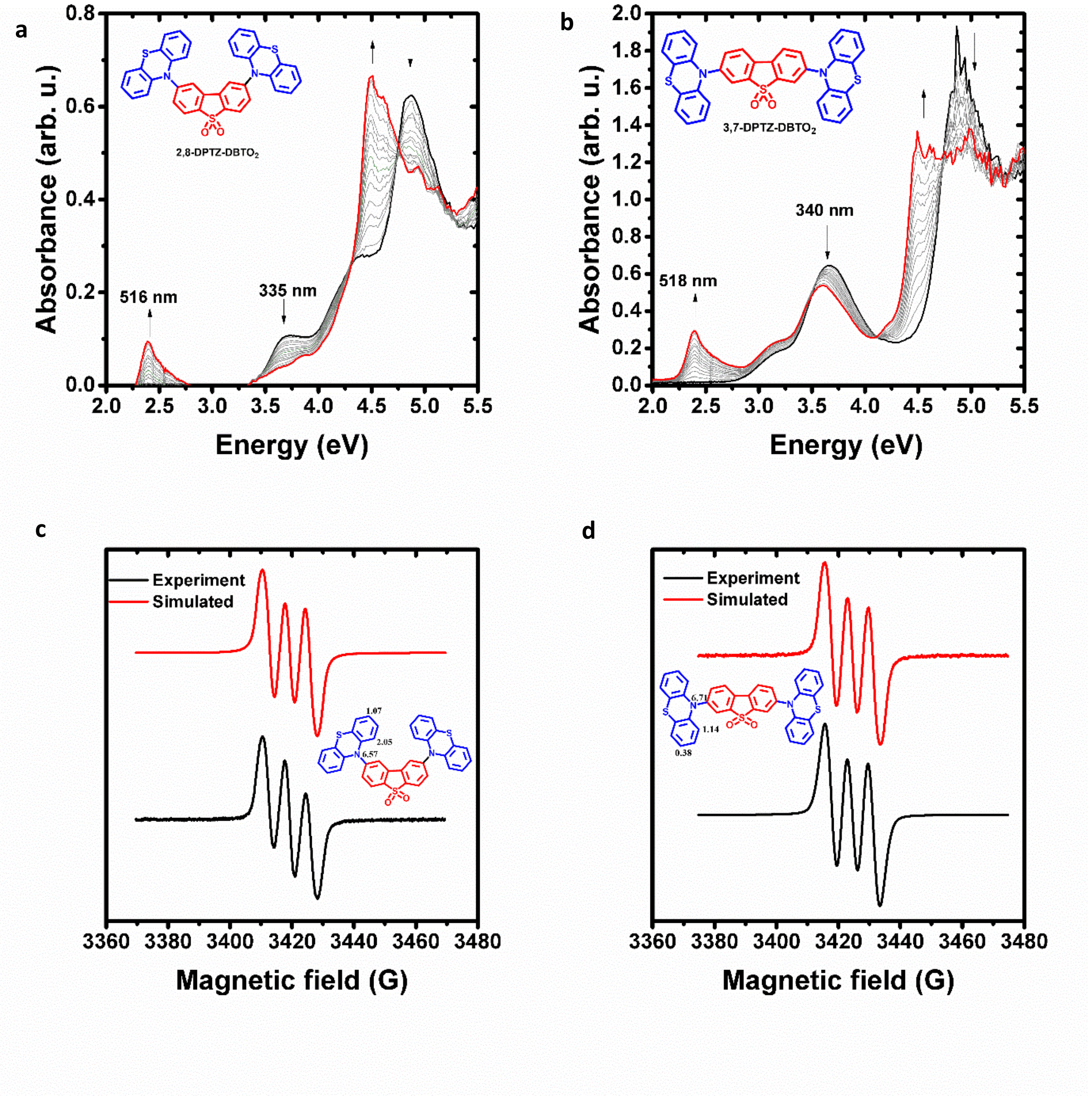

Supplementary Figure 2| UV-Vis and EPR spectroelectrochemical measurement of oxidation process of the isomers. UV-Vis spectroelectrochemical measurement of oxidation process in (**a**) 2,8-DPTZ-$DBTO_2$ and (**b**) 3,7-DPTZ-$DBTO_2$. EPR spectra of electrochemically generated radical cations registered in 0.1M Bu_4NBF_4 dichloromethane solution at potential 0.6 V for (**c**) 2,8-DPTZ-$DBTO_2$ and 0.5 V for (**d**) 3,7-DPTZ-$DBTO_2$, with simulated spectra (red).

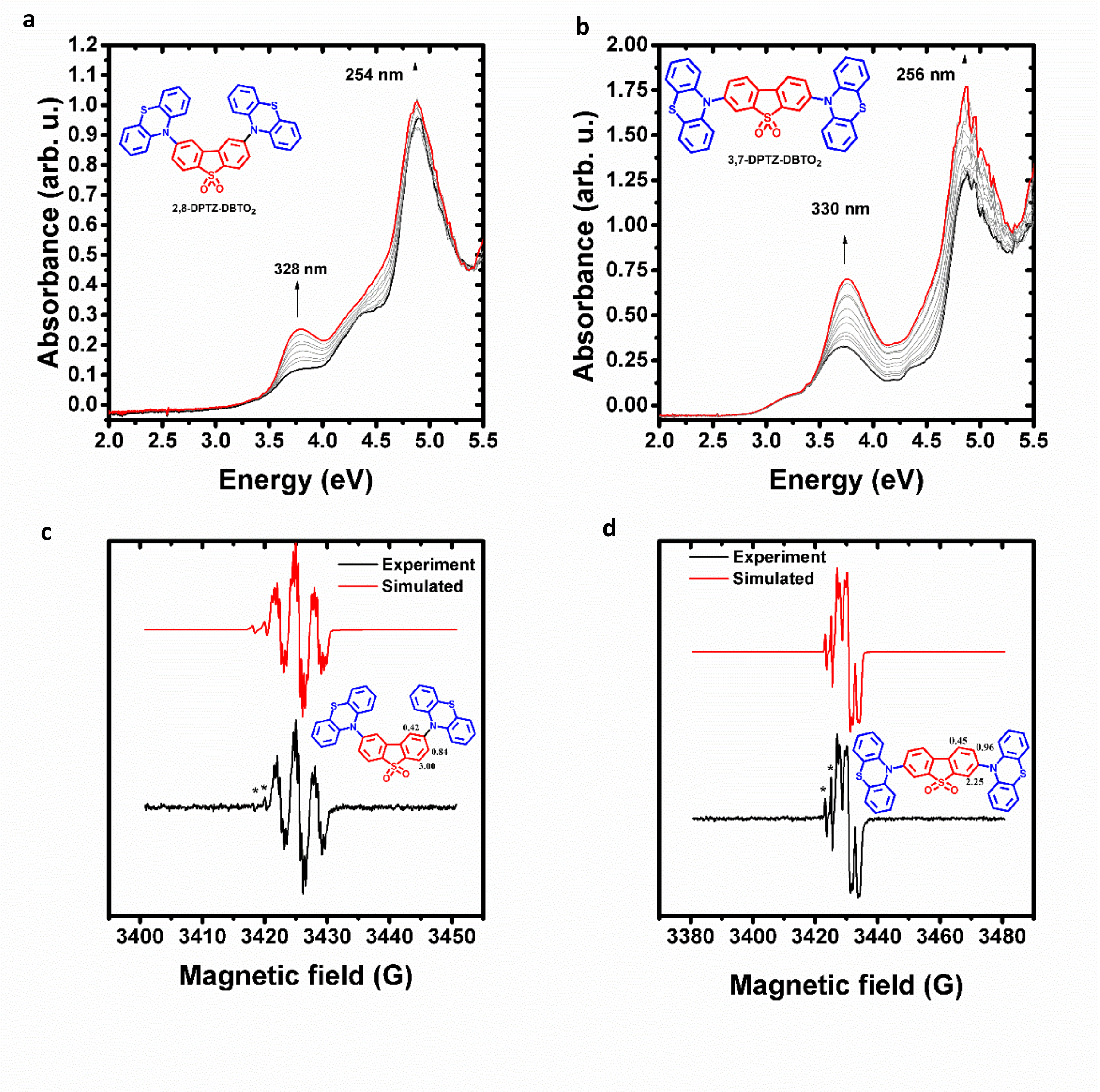

Supplementary Figure 3| UV-Vis and EPR spectroelectrochemical measurements of the reduction process of the isomers. UV-Vis spectroelectrochemical measurement of reduction process of (**a**) 2,8-DPTZ-DBTO$_2$, and (**b**) 3,7-DPTZ-DBTO$_2$. EPR spectra of electrochemically generated radical anions registered in 0.1M Bu_4NBF_4 dichloromethane solution at potential -2.3 V for (**c**) 2,8-DPTZ-DBTO$_2$ and (**d**) 3,7-DPTZ-DBTO$_2$, with simulated spectra (red).

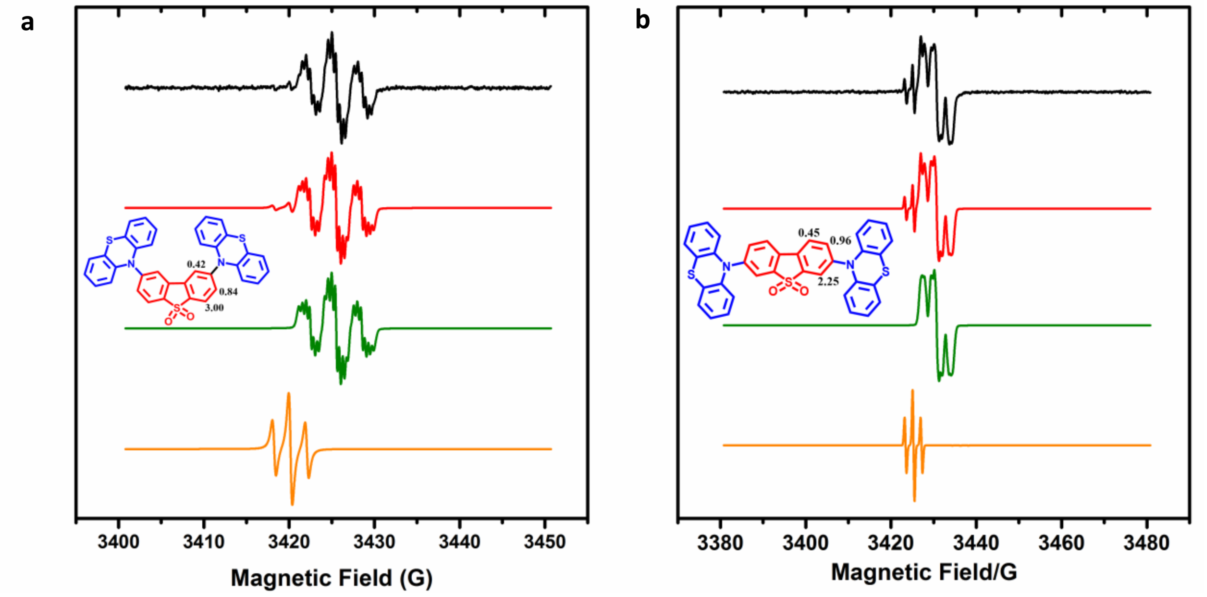

Supplementary Figure 4| Distribution of simulated spectra of the isomers. Simulated spectra of (**a**) 2,8-DPTZ-DBTO$_2$ and (**b**) 3,7-DPTZ-DBTO$_2$. EPR measured signal (black line), simulated signal of two radicals (red line), simulated signal of first radical at first doping stage (green line), simulated signal of second radical at second doping stage (orange line).

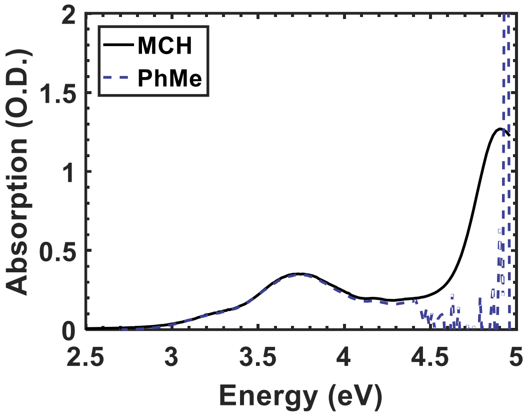

Supplementary Figure 5| 3,7-DPTZ-DBTO$_2$ absorption as a function of solvent polarity

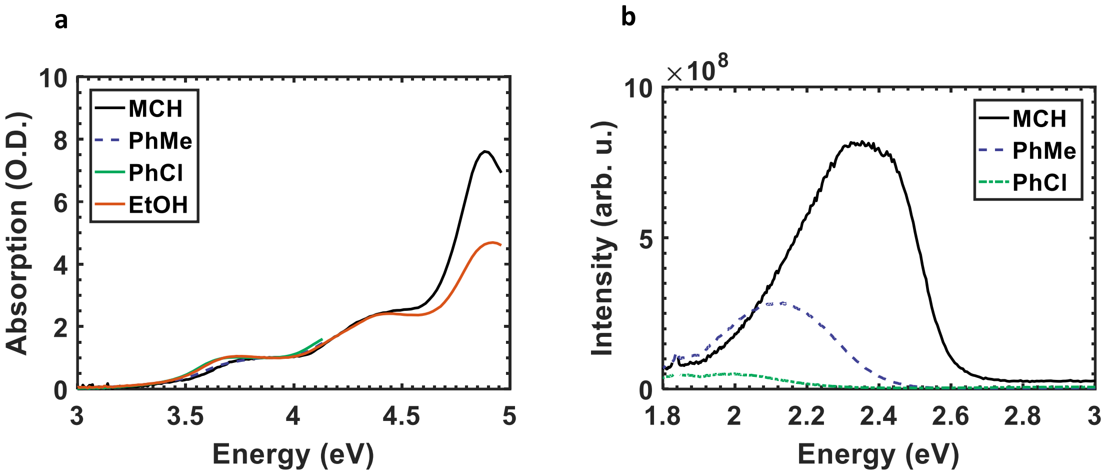

Supplementary Figure 6| The effect of solvent polarity on the absorption and emission of 2,8-DPTZ-DBTO$_2$. (**a**) The ground-state absorption of 2,8-DPTZ-DBTO$_2$ as a function of solvent polarity. (**b**) The emission of 2,8-DPTZ-DBTO$_2$ as a function of solvent polarity showing a clear bathochromic shift.

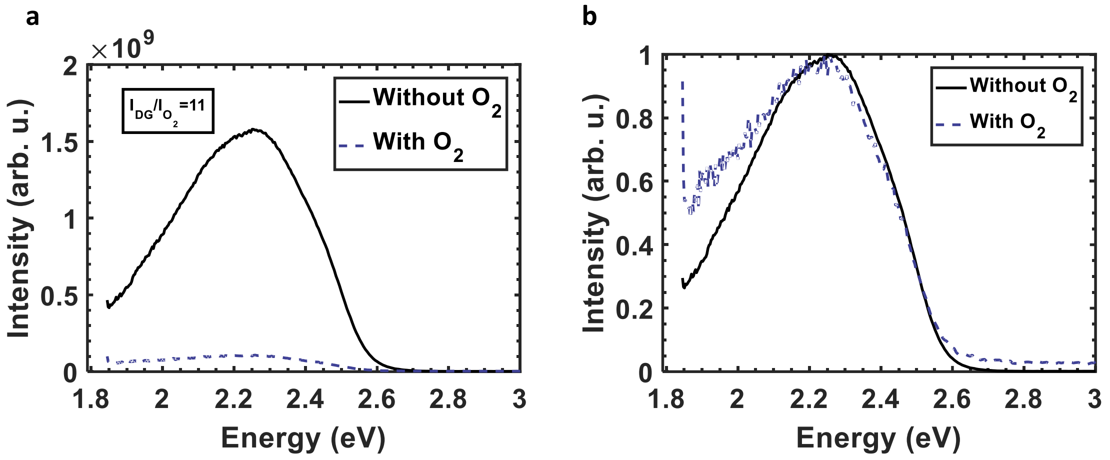

Supplementary Figure 7| Degassed versus non-degassed solutions of 2,8-DPTZ-DBTO$_2$ in MCH (**a**) Comparison of intensities of degassed (black solid line) versus non-degassed (blue dashed line) indicating the ratio of 11 between the two conditions. (**b**) The normalised emissions indicate that they are the same spectral shape.

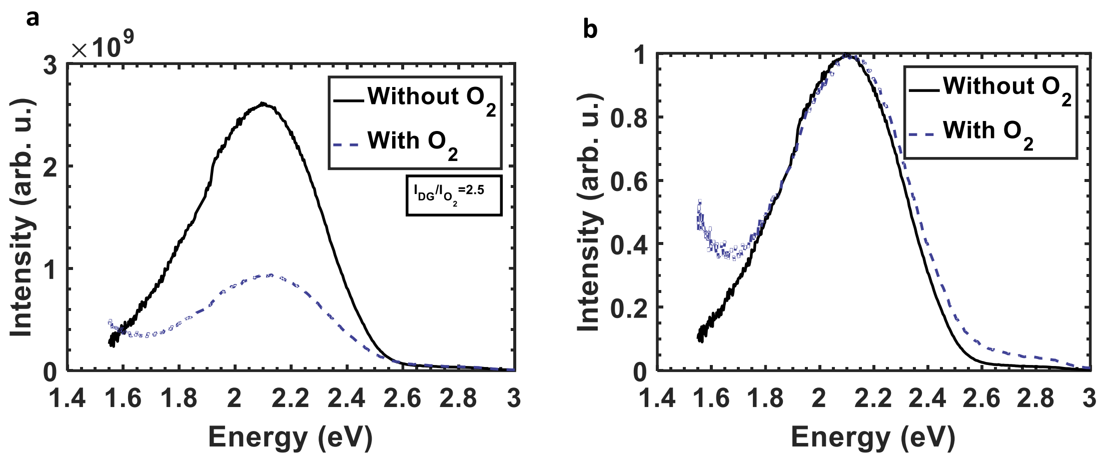

Supplementary Figure 8| Degassed versus non-degassed solutions of 3,7-DPTZ-DBTO$_2$ in MCH (**a**) Comparison of intensities of degassed (black solid line) versus non-degassed (blue dashed line) indicating the ratio of 2.5 between the two conditions. (**b**) The normalised emissions indicate that they are the same spectral shape

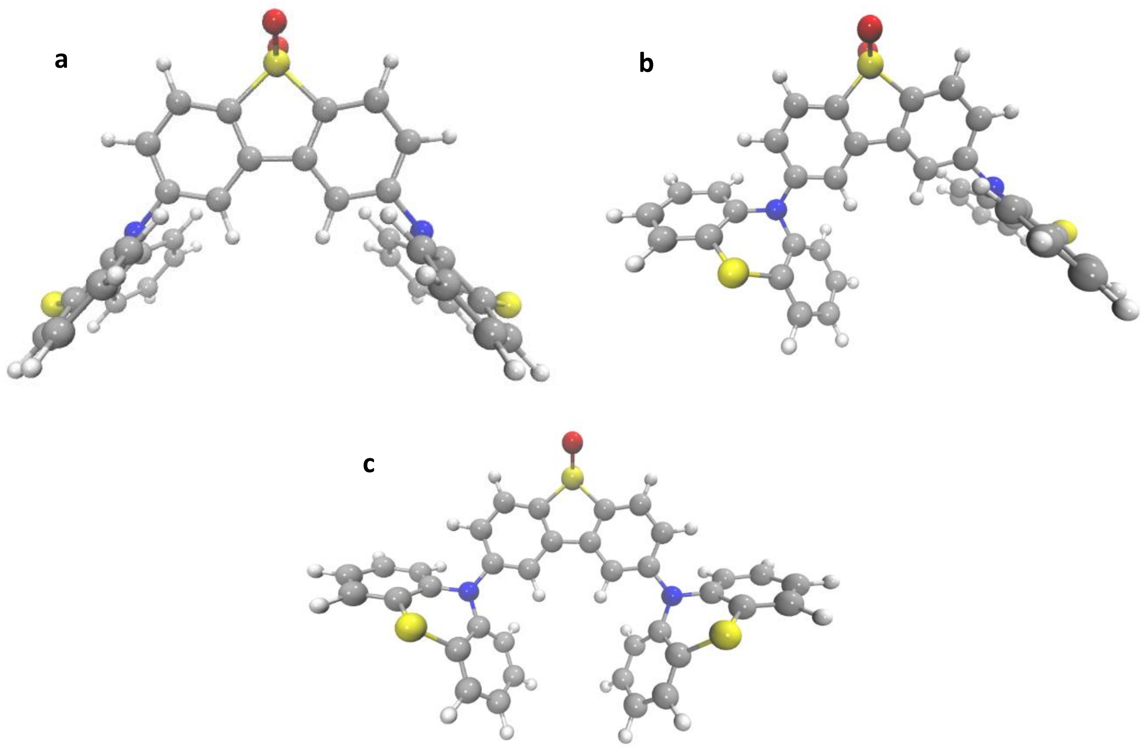

Supplementary Figure 9| Possible conformations of the 2,8-DPTZ-$DBTO_2$ isomer (**a**) ax-ax (**b**) eq-ax (**c**) eq-eq.

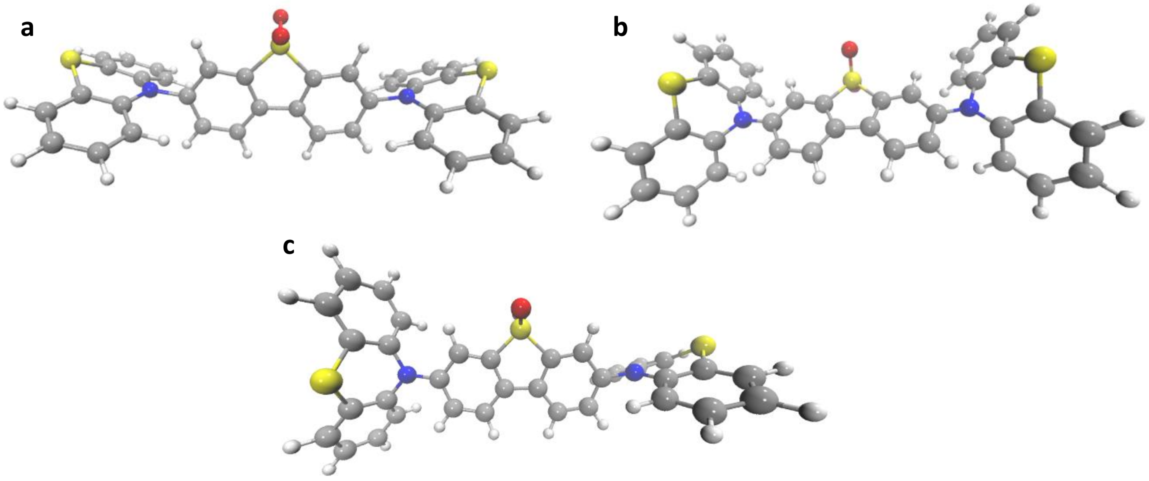

Supplementary Figure 10| Possible conformations of the 3,7-DPTZ-DBTO$_2$ isomer (**a**) ax-ax (**b**) eq-eq (**c**) eq-ax

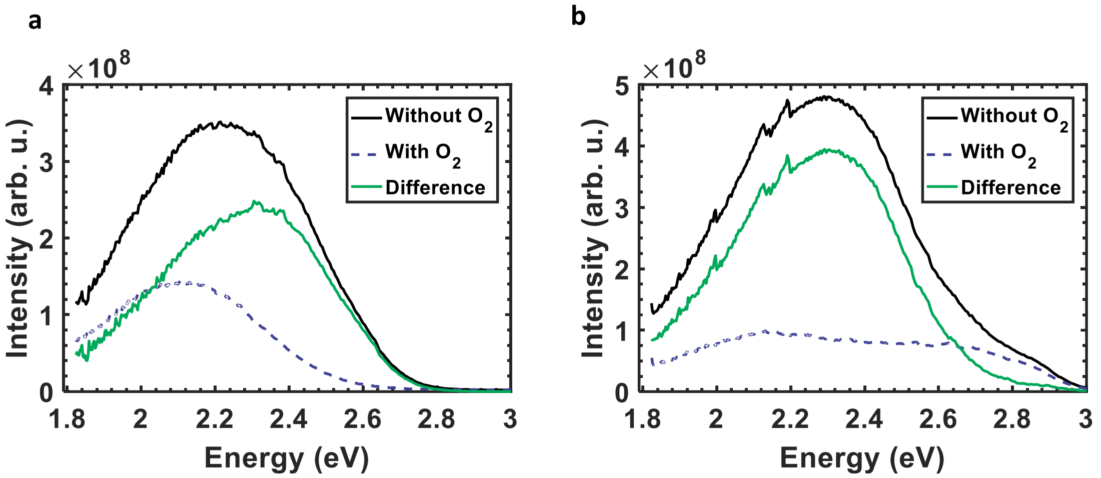

Supplementary Figure 11|Comparison of the degassed and non-degassed emission of the isomers in a zeonex host. (**a**) Emission of 2,8-DPTZ-$DBTO_2$ in zeonex as a function of oxygen. The difference between the two spectra clearly shows higher onset emission than ^{1}CT which is attributed to ^{3}LE. (**b**) Emission of 3,7-DPTZ-$DBTO_2$ in zeonex as a function of oxygen with the CT_{ax} emission observed at onset ca. 3 eV and also the contribution of ^{3}LE giving a higher onset than the expected 2.50 eV for CT_{eq}.

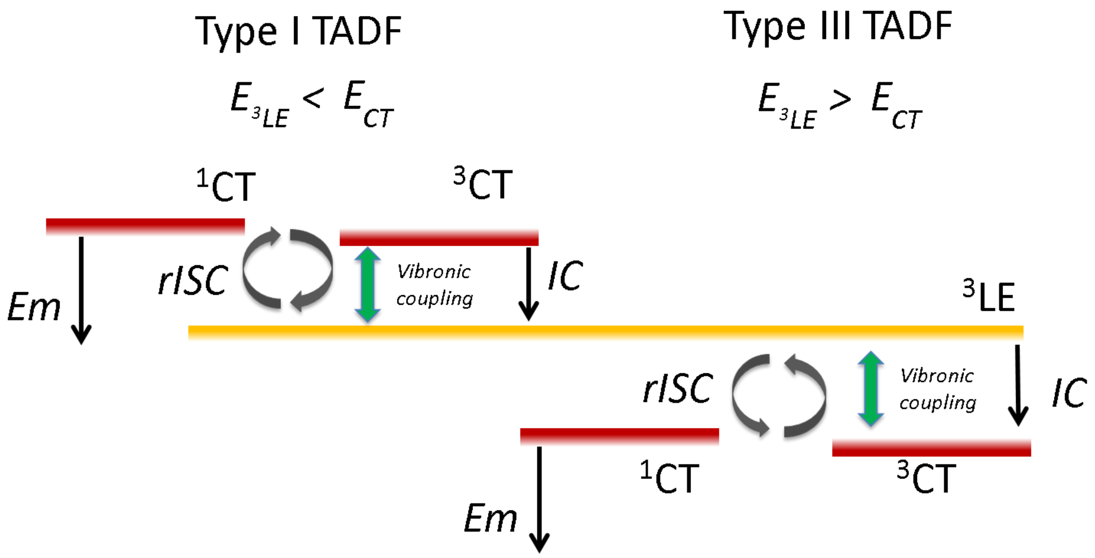

Supplementary Figure 12|Energy level schematic for the TADF mechanism. Two energy level orderings for the TADF mechanism showing Type I, where ^{1}CT>^{3}LE, on the left-hand side (LHS) and Type III, where ^{1}CT<^{3}LE, on the right-hand side (RHS). These mechanisms are presented completely in previous work; from which we use the notation.[1] Type II TADF is where the ^{3}LE and ^{1}CT are considered isoenergetic.

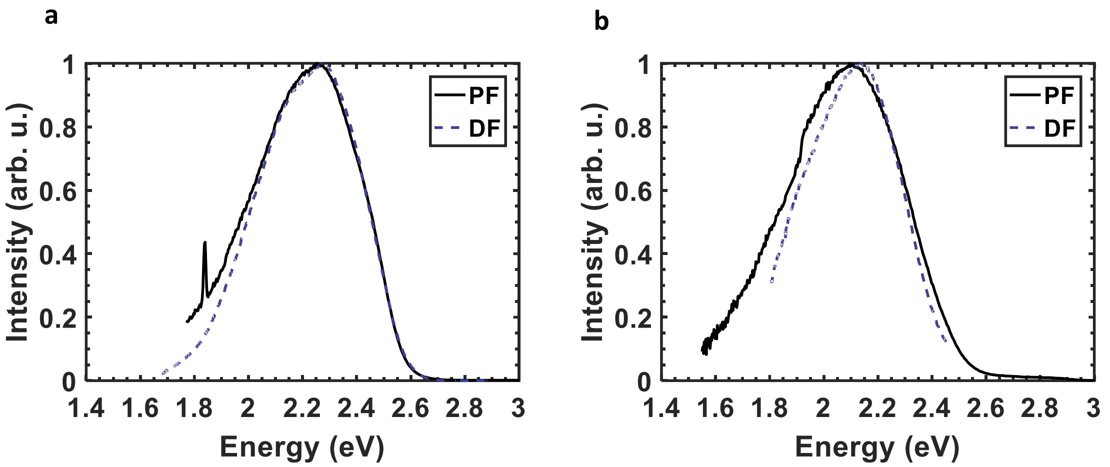

Supplementary Figure 13| Comparison of prompt fluorescence and delayed fluorescence of 2,8-DPTZ-DBTO$_2$ and 3,7-DPTZ-DBTO$_2$ in MCH. (**a**) The prompt fluorescence (PF) and delayed fluorescence (DF) of 2,8-DPTZ-DBTO$_2$ match in terms of their spectral shape. (**b**) This is similarly shown for 3,7-DPTZ-DBTO$_2$.

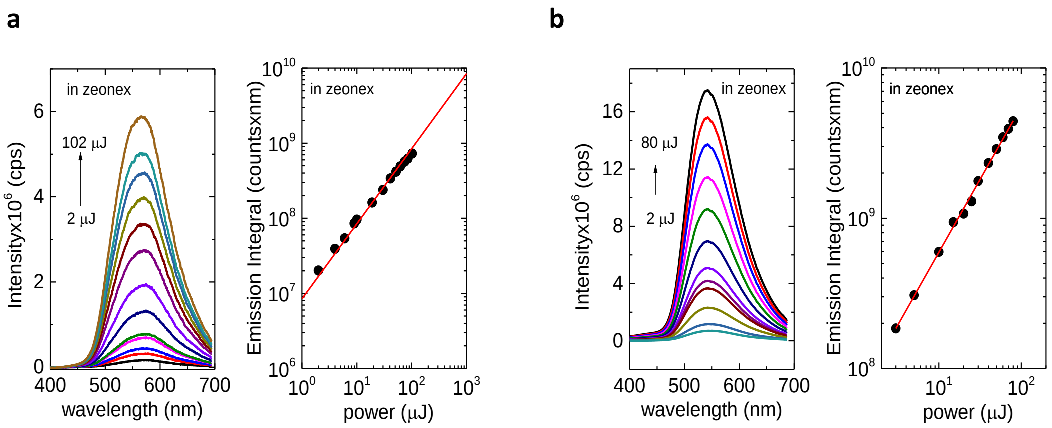

Supplementary Figure 14| Excitation intensity dependence of 2,8-DPTZ-DBTO$_2$ and 3,7-DPTZ-DBTO$_2$ in zeonex films. (**a**) The change in spectra of 2,8-DPTZ-DBTO$_2$ with increasing intensity and the linear power dependence on intensity indicating TADF. (**b**) Similarly for 3,7-DPTZ-DBTO$_2$. The spectra are plotted as a function of wavelength and have not been corrected using the Jacobian found in Mooney and Kambhampati,[2] as the integral of the emission versus power is the focus. Spectra were acquired with 1 μs delay time and integrated for 200 μs.

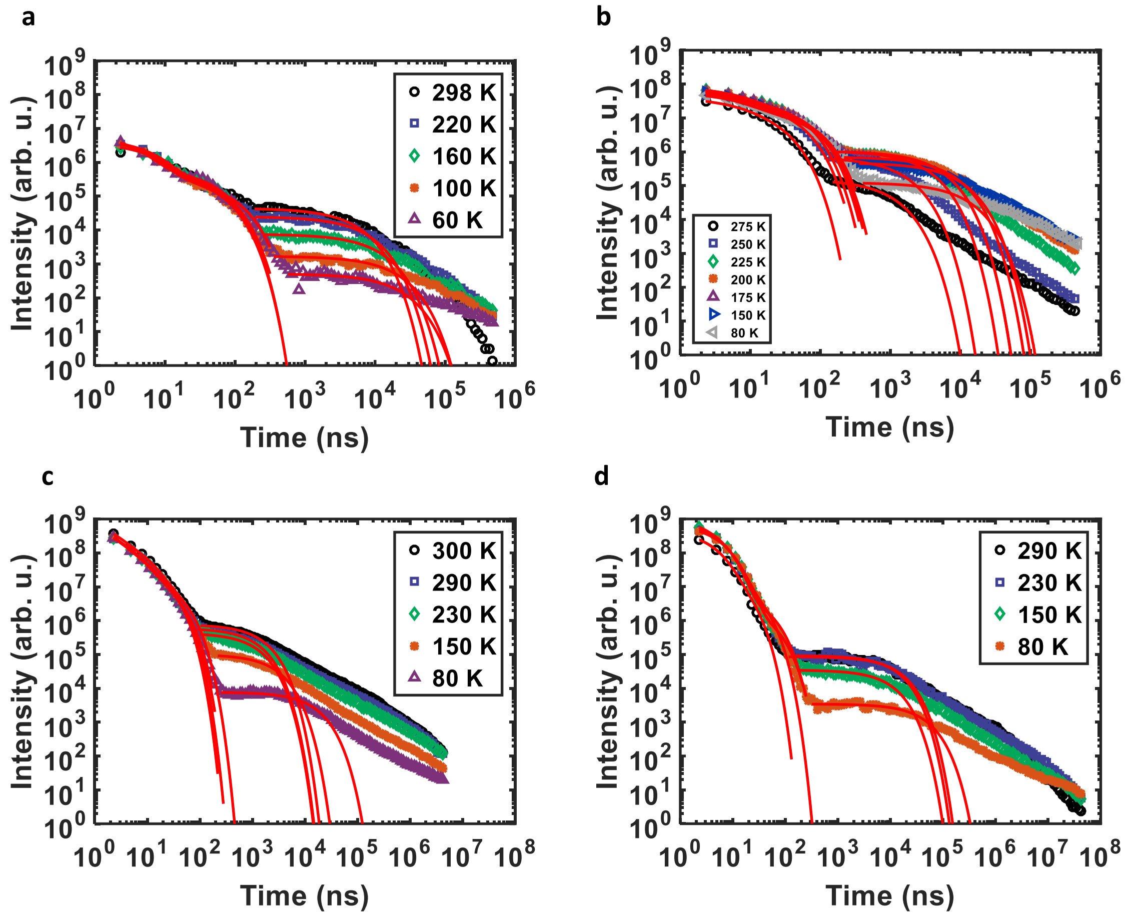

Supplementary Figure 15| Emission decay profiles of a 2,8-DPTZ-DBTO$_2$ and 3,7-DPTZ-DBTO$_2$ in a variety of solid state hosts as a function of temperature. (**a**) **2,8-DPTZ-DBTO$_2$** in CBP where the rigidity of the matrix prevents change in the ΔE_{S-T} gap and thus prompt CT_{eq} lifetime (<10^2 ns) remains unchanged. (**b**) 2,8-DPTZ-DBTO$_2$ in polyethylene oxide (PEO), which has a diminishing ΔE_{S-T} gap with increasing rigidity and decreasing temperature, thus the prompt CT_{eq} lifetime increases with decreasing temperature. (**c**) 3,7-DPTZ-DBTO$_2$ in CBP, where the rigidity of the matrix prevents change in the ΔE_{S-T} gap and the lifetime remains unchanged. (**d**) 3,7-DPTZ-DBTO$_2$ in Zeonex, which is more rigid than PEO but less rigid than CBP and as such there is minimal change in ΔE_{S-T} and thus prompt CT_{eq} lifetime. The red lines are the exponential fits to the data, the parameters of which can be found in Supplementary Tables 10-13.

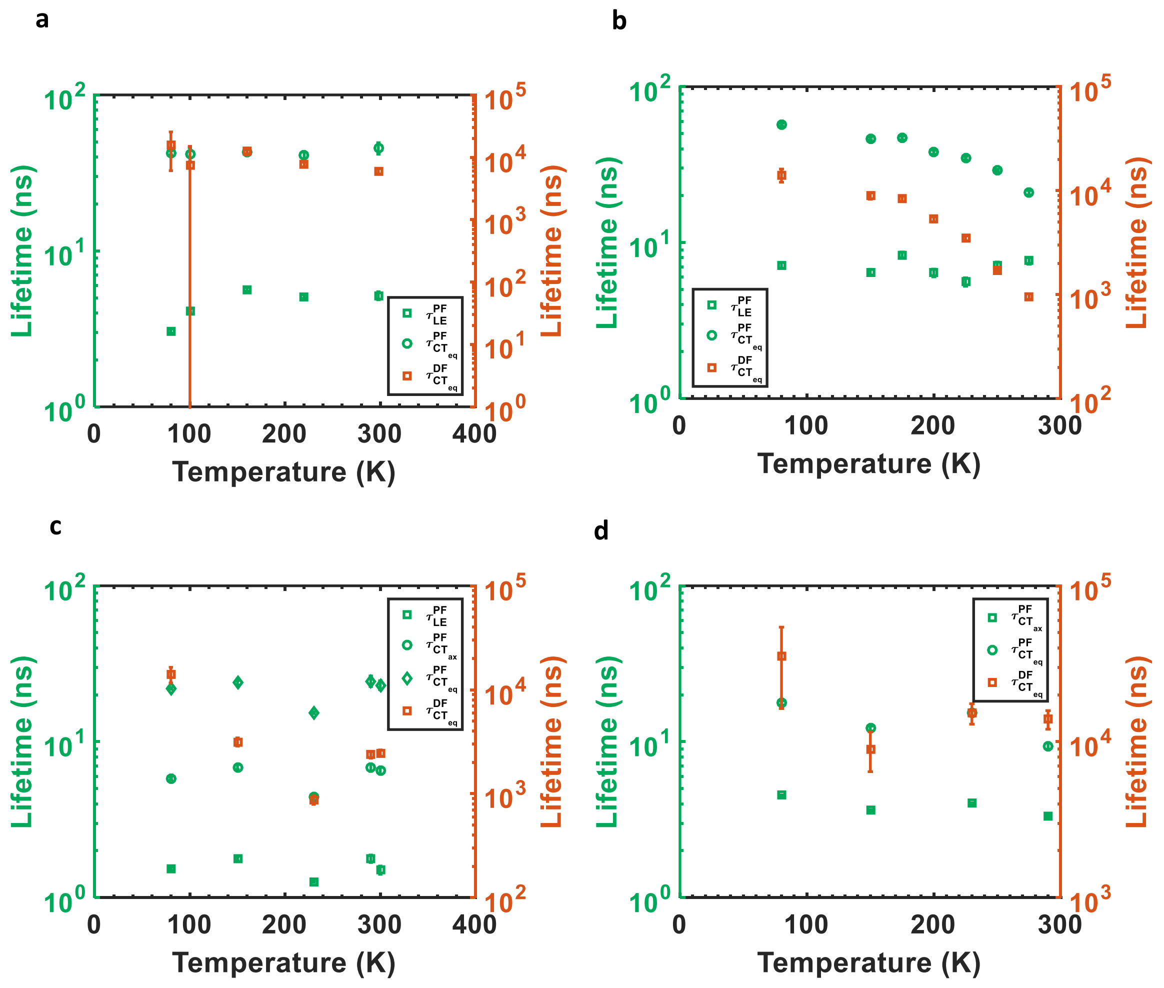

Supplementary Figure 16| Decay lifetimes of 2,8-DPTZ-DBTO$_2$ and 3,7-DPTZ-DBTO$_2$ in a variety of solid state hosts as a function of temperature. (**a**) **2,8-DPTZ-DBTO$_2$** in CBP, where the rigidity of the matrix means that there is no change in the energy gap and thus no change in lifetime. (The large error bar at 100 K on the DF is due to a small amplitude in the decay, however this does not detract from the trend.) (**b**) **2,8-DPTZ-DBTO$_2$** in PEO, where the change in energy gap with temperature means that there is an increase in the lifetime of the prompt and delayed CT$_{eq}$ emission. This agrees with the resonance curves published in previous work.[1] The prompt LE lifetime remains unchanged however, as this is not affected by rISC rate. (**c**) 3,7-DPTZ-DBTO$_2$ in CBP, where the rigidity of the matrix again prevents any change in the lifetime. The variation in the delayed fluorescence lifetime of CT$_{eq}$ is due to the early onset of a power law decay in the data. This can skew the exponential fit and thus the uncertainty in those values is much larger than the error bars. (**d**) 3,7-DPTZ-DBTO$_2$ in zeonex, where even though the matrix is not as rigid as CBP there is very minimal change in the lifetimes of the species. The error bars are the error on the fit and do not consider any uncertainty in whether the decay is exponential or power law.

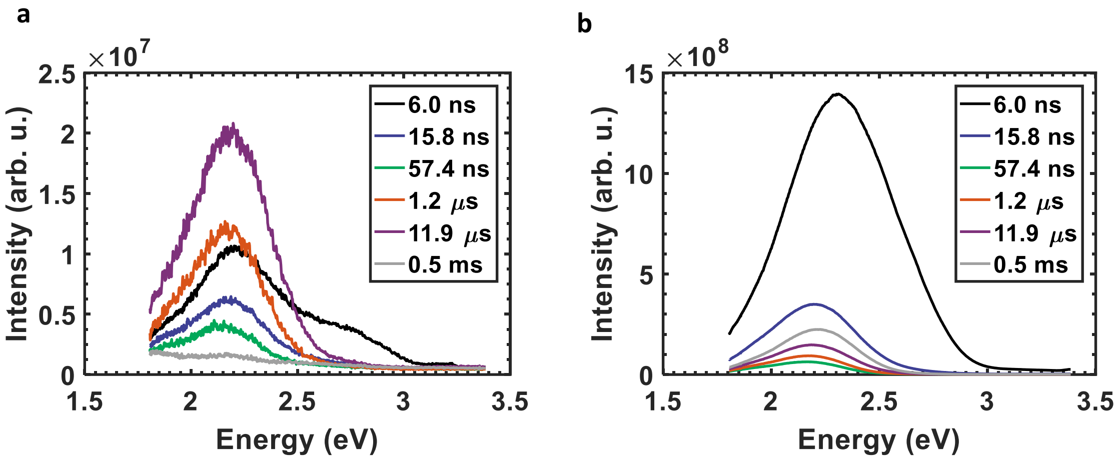

Supplementary Figure 17| Time-resolved emission spectra of 2,8-DPTZ-DBTO$_2$ and 3,7-DPTZ-DBTO$_2$ in CBP (**a**) The time-resolved (TR) spectra of 2,8-DPTZ-DBTO$_2$ in CBP shows ^{1}LE character at early times, which then becomes completely $^1CT_{eq}$ by 15 ns onwards. (**b**) The TR spectra of 3,7-DPTZ-DBTO$_2$ in CBP shows a combination of $^1CT_{ax}$ and $^1CT_{eq}$ that becomes purely $^1CT_{eq}$. This is indicated by the spectral shift to lower energies within 10 ns, and is corroborated in the emission decay fits (Supplementary Table 10).

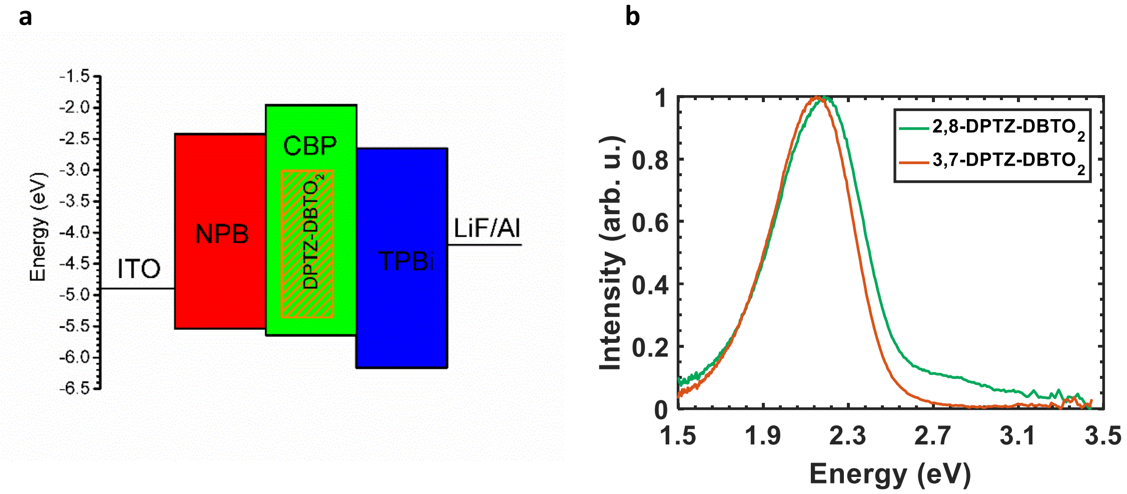

Supplementary Figure 18| The structure and electroluminescence of 2,8-DPTZ-DBTO$_2$ and 3,7-DPTZ-DBTO$_2$ in devices. (**a**) The typical device structure used for the 2,8-DPTZ-DBTO$_2$ and 3,7-DPTZ-DBTO$_2$ devices. (**b**) The electroluminescence spectra of the 2,8-DPTZ-DBTO$_2$ and 3,7-DPTZ-DBTO$_2$ devices showing that as in the photoluminescence 3,7-DPTZ-DBTO$_2$ is still more red-shifted in emission than 2,8-DPTZ-DBTO$_2$.

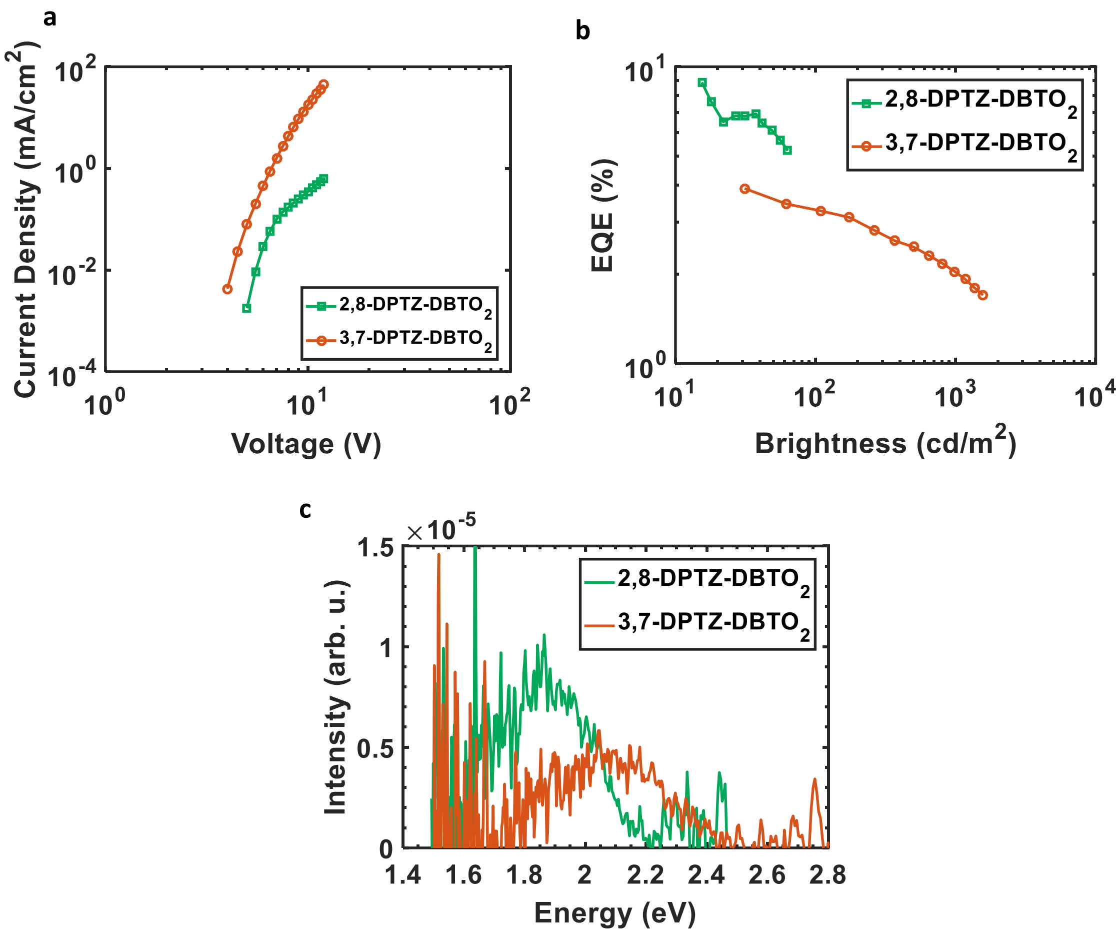

Supplementary Figure 19| Device characteristics of the isomers in a DPEPO host device. (**a**) The J-V curves of the devices. (**b**) EQE vs brightness for the devices. (**c**) Comparison of the electroluminescence for the devices at the same current density.

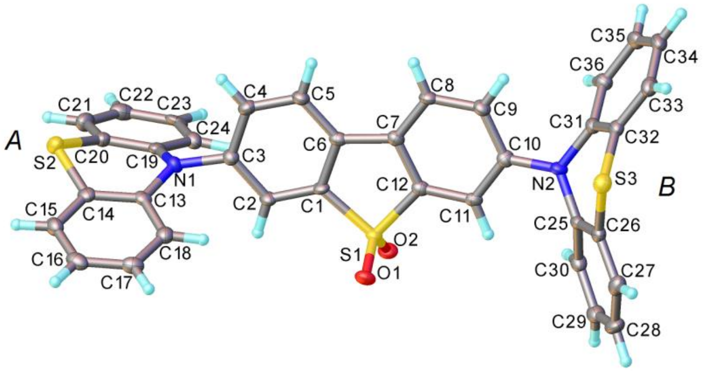

Supplementary Figure 20| Molecular structure of 3,7-DPTZ-DBTO$_2$. Displacement ellipsoids are drawn at the 50% probability level.

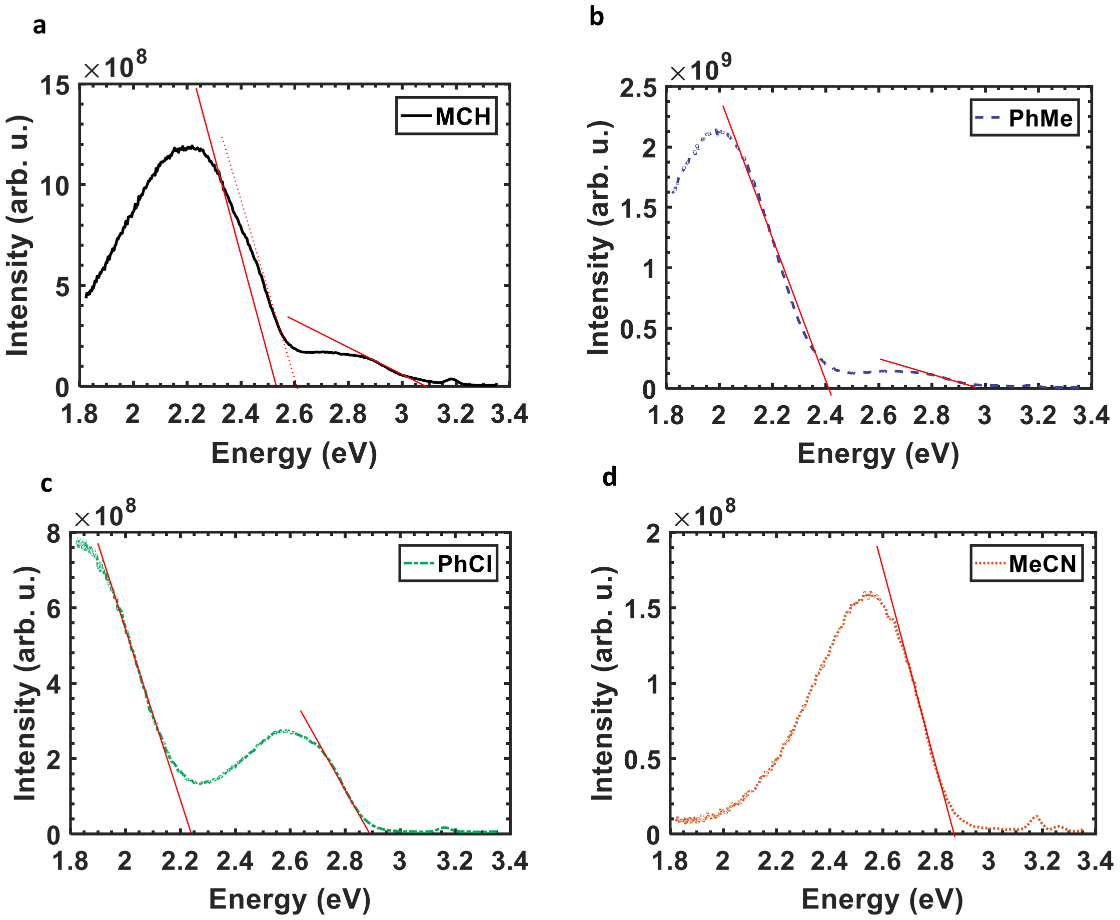

Supplementary Figure 21 | Measurement of charge-transfer state onset energies of 3,7-DPTZ-DBTO$_2$ **(a)** Measurement of quasi-axial and quasi-equatorial CT state onsets (red dashed lines) in MCH, there is also indicated an extra shoulder (dotted line) which is assigned to overlap of the two CT states. **(b)** Measurement of quasi-axial and quasi-equatorial CT state onsets in PhMe. **(c)** Measurement of quasi-axial and quasi-equatorial CT state onsets in PhCl. **(d)** Measurement of quasi-axial CT state onset in MeCN. This method was also used in our previous work[1] and can be found in the Supporting Information of that article. Extracted energies using this method can be found in Supplementary Table 15.

Supplementary Table 1| Electrochemical Ionization Potential (IP) and Electron Affinity (EA) energies

Compound	IP - HOMO (eV)	EA - LUMO (eV)
**2,8-DPTZ-DBTO$_2$**	−5.45	−3.05
**3,7-DPTZ-DBTO$_2$**	−5.40	−3.05

Supplementary Table 2| Spectroscopic properties of investigated compounds in neutral and in oxidized states.

	Neutral State		Absorption bands (nm)				g-value
Compound	λ_{max} (nm)	E_g^{opt} (eV)	Oxidized state				
2,8-DPTZ-$DBTO_2$	336	3.37	276		516		2.00549
3,7-DPTZ-$DBTO_2$	396	2.82	276	345	396	518	2.00565

Supplementary Table 3| Spectroscopic properties of investigated compounds in reduced state.

	Absorption bands (nm)		g-value
Compound	Reduced state		
2,8-DPTZ-$DBTO_2$	254	328	2.00238
3,7-DPTZ-$DBTO_2$	254	329	2.00236

Supplementary Table 4| Relative energies of the 3 conformers of 2,8-DPTZ-DBTO$_2$ and 3,7-DPTZ-DBTO$_2$ and the prevalence of each structure calculated using a Boltzmann distribution. The energetics have been calculated using a DFT(PBE0) optimised geometry and a polarisable continuum model with the dielectric constant of water.

	Relative Energy (eV)	% conformer
**2,8-DPTZ-DBTO$_2$ axial-axial**	0.38	0.00
**2,8-DPTZ-DBTO$_2$ axial-equatorial**	0.07	0.05
**2,8-DPTZ-DBTO$_2$ equatorial - equatorial**	0.00	0.95

**3,7-DPTZ-DBTO$_2$ axial-axial**	0.09	0.02
**3,7-DPTZ-DBTO$_2$ axial-equatorial**	0	0.93
**3,7-DPTZ-DBTO$_2$ equatorial - equatorial**	0.07	0.05

Supplementary Table 5| The exponential decay parameters of the two isomers in MCH and Toluene solution at room temperature

Material (Solvent)	A_{CT}^{PF}	τ_{CT}^{PF} (ns)	A_{CT}^{PF}	τ_{CT}^{PF} (ns)
2,8-DPTZ-$DBTO_2$ (MCH)	1.02±0.02E4	15.1±0.3	270±8	5.4±0.3E3
2,8-DPTZ-$DBTO_2$ (Toluene)	1.05±0.02E4	32.9±0.7	172±17	1.1±0.1E3
3,7-DPTZ-$DBTO_2$ (MCH)	2.43±0.03E8	7.31±0.07	5.6±0.9E4	3.7±1.1E3
3,7-DPTZ-$DBTO_2$ (Toluene)	9.4±0.1E8	15.9±0.2	1.6±0.2E6	2.8±0.5E3

Supplementary Table 6| Parameters for the 3 state D-A model Hamiltonian of 2,8-DPTZ-DBTO$_2$ described above. These have been previously published in ref.[3]

Parameter	Value (cm^{-1})
ω_1	13.61
ω_{11}	173.65
ω_{23}	422.60
E_{SOC}	2.00
E_{HFI}	0.20
Relative Energy ^3LE_D	0.00
Relative Energy ^3CT	0.01
Relative Energy ^1CT	0.04
λ_1	67.02 (^3CT-^3LE_D)
λ_{11}	91.71 (^3CT-^3LE_D)
λ_{23}	79.04 (^3CT-^3LE_D)

Supplementary Table 7| Parameters for the 4 state D-A model Hamiltonian of 2,8-DPTZ-DBTO$_2$ Hamiltonian described above.

Parameter	Value (cm^{-1})
ω_1	13.61
ω_5	84.60
ω_9	128.32
ω_{11}	173.65
ω_{23}	422.60
Relative Energy ^3LE_D	0.00
Relative Energy ^3LE_A	0.14
Relative Energy ^3CT	0.02
Relative Energy ^1CT	0.05
E_{SOC}	2.00
E_{HFI}	0.20
λ_1	67.02 (^3CT – ^3LE_D) 143.00 (^3CT – ^3LE_A)
λ_{11}	91.71 (^3CT – ^3LE_D) 75.65 (^3CT – ^3LE_A)
λ_{23}	79.04 (^3CT – ^3LE_D) 75.65 (^3CT – ^3LE_A)
λ_5	91.78 (^3CT – ^3LE_A)
λ_9	183.89 (^3LE_D – ^3LE_A)

Supplementary Table 8| Parameters for the 3 state D-A model Hamiltonian of 3,7-DPTZ-DBTO$_2$ described above.

Parameter	Value (cm^{-1})
ω_2	13.61
ω_9	173.65
ω_{12}	422.60
Relative Energy ^3LE_D	0.06
Relative Energy ^3CT	0.00
Relative Energy ^1CT	0.03
E_{SOC}	2.00
E_{HFI}	0.20
λ_2	216.07 (^3CT – ^3LE_D)
λ_9	112.75 (^3CT – ^3LE_D)
λ_{12}	169.53 (^3CT – ^3LE_D)

Supplementary Table 9| The exponential decay parameters of the two isomers in a zeonex matrix at room temperature

Material	A_{LE}^{PF}	τ_{LE}^{PF} (ns)	$A_{CT_{eq}}^{PF}$	$\tau_{CT_{eq}}^{PF}$ (ns)	$A_{CT_{eq}}^{DF}$	$\tau_{CT_{eq}}^{DF}$ (ns)
2,8-DPTZ-$DBTO_2$	2.08±0.07E8	3.3±0.1	1.4±0.1E7	24.5±1.2	8.3±0.3E5	7.4±0.5E3
	$A_{CT_{ax}}^{PF}$	$\tau_{CT_{ax}}^{PF}$ (ns)	$A_{CT_{eq}}^{PF}$	$\tau_{CT_{eq}}^{PF}$ (ns)	$A_{CT_{eq}}^{DF}$	$\tau_{CT_{eq}}^{DF}$ (ns)
3,7-DPTZ-$DBTO_2$	4.24±0.05E8	3.32±0.06	3.9±0.5E7	9.4±0.4	9.0E4±0.5E4	1.4±0.2E4

Supplementary Table 10| The exponential decay fitting parameters of the 3,7-DPTZ-DBTO$_2$ in zeonex film time-resolved emission

Temperature (K)	$A^{PF}_{CT_{ax}}$	$\tau^{PF}_{CT_{ax}}$ (ns)	$A^{PF}_{CT_{eq}}$	$\tau^{PF}_{CT_{eq}}$ (ns)	$A^{DF}_{CT_{eq}}$	$\tau^{DF}_{CT_{eq}}$ (ns)
290	4.24±0.04E8	3.32±0.06	3.9±0.5E7	9.4±0.4	9.0±0.5E4	1.3±0.2E4
230	8.22±0.05E8	4.02±0.03	2.6±0.2E7	15.3±0.6	8.7E4±0.6E4	1.5±0.2E4
150	9.85±0.07E8	3.62±0.04	5.2±0.5E7	12.2±0.4	3.9±0.5E4	9.0±0.3E4
80	6.77±0.03E8	4.53±0.03	2.1±0.1E7	17.7±0.5	3.9±0.8E3	3.5±1.9E4

Supplementary Table 11| The exponential decay fitting parameters of the 2,8-DPTZ-DBTO$_2$ in CBP film time-resolved emission

Temperature (K)	A_{LE}^{PF}	τ_{LE}^{PF} (ns)	$A_{CT_{eq}}^{PF}$	$\tau_{CT_{eq}}^{PF}$ (ns)	$A_{CT_{eq}}^{DF}$	$\tau_{CT_{eq}}^{DF}$ (ns)
298	4.3±0.3E6	5.2±0.3	4.2±0.4E5	46±4	3.9±0.2E4	6.0±0.6E3
220	4.97±0.09E6	5.1±0.1	4.4±0.3E5	41±2	2.3±0.1E4	7.7±0.6E3
160	3.44±0.07E6	5.7±0.2	5.0±0.2E5	43±1	6.8±0.4E3	1.2±0.2E4
100	5.32±0.06E6	4.13±0.09	4.5±04E5	42±4	2.3±5.3E3	7.5±7.5E3
80	6.8±0.2E6	3.06±0.09	6.1±0.2E5	43±1	6.0±1.6E2	1.6±1.0E4

Supplementary Table 12| The exponential decay fitting parameters of the 3,7-DPTZ-DBTO$_2$ in CBP film time-resolved emission

Temperature (K)	A_{LE}^{PF}	τ_{LE}^{PF} (ns)	$A_{CT_{ax}}^{PF}$	$\tau_{CT_{ax}}^{PF}$ (ns)	$A_{CT_{eq}}^{PF}$	$\tau_{CT_{eq}}^{PF}$ (ns)	$A_{CT_{eq}}^{DF}$	$\tau_{CT_{eq}}^{DF}$ (ns)
300	8.3±0.5E8	1.50±0.09	2.3±0.1E8	6.5±0.3	2.3±0.4E7	23±2	5.9±0.2E5	2.5±0.2E3
290	5.2±0.2E8	1.78±0.10	1.6±0.1E8	6.8±0.4	1.3±0.2E7	25±2	4.1±0.2E5	2.4±0.2E3
230	6.80±0.06E8	1.27±0.02	2.26±0.03E8	4.46±0.06	4.3±0.1E7	15.4±0.2	4.9±0.2E5	874±85
150	6.20±0.1E8	1.78±0.05	1.36±0.06E8	6.9±0.2	1.7±0.1E7	23.9±0.8	9.5±0.5E4	3.2±0.3E3
80	7.1±0.1E8	1.53±0.03	1.58±0.05E8	5.8±0.1	2.12±0.08E7	22.0±0.3	8.0±0.6E3	1.4±0.2E4

Supplementary Table 13| The exponential decay fitting parameters of the 2,8-DPTZ-DBTO$_2$ in PEO film time-resolved emission

Temperature (K)	A_{LE}^{PF}	τ_{LE}^{PF} (ns)	$A_{CT_{eq}}^{PF}$	$\tau_{CT_{eq}}^{PF}$ (ns)	$A_{CT_{eq}}^{DF}$	$\tau_{CT_{eq}}^{DF}$ (ns)
275	2.4±0.1E7	7.6±0.4	1.4±0.1E7	20.9±0.7	1.44±0.07E5	958±59
250	2.73±0.09E7	7.1±0.4	2.95±0.09E7	29.0±0.5	5.5±0.1E5	1.70±0.6E3
225	5.0±0.2E7	5.6±0.3	3.3±0.1E7	34.9±0.8	9.1±0.3E5	3.5±0.2E3
200	5.2±0.2E7	6.4±0.4	3.0±0.1E7	38±1	9.1±0.3E5	5.3±0.3E3
175	4.9±0.1E7	8.4±0.4	2.19±0.09E7	47±1	6.2±0.2E5	8.4±0.5E3
150	5.1±0.1E7	6.5±0.3	2.17±0.07E7	46±1	4.6±0.2E5	8.9±0.6E3
80	3.88±0.09E7	7.2±0.3	1.65±0.05E7	57±1	1.12±0.08E5	1.4±0.2E4

Supplementary Table 14| Computational details for the MCTDH simulations. N_i is the number of primitive harmonic oscillator discrete variable representation (DVR) basis functions used to describe each mode. n_i is the number of single-particle functions used to describe the wavepacket.

Simulation	Modes	N_i	n_i
**ISC 2,8-DPTZ-DBTO$_2$**	v1	21	12,12,12
**(3 state)**	v11,v23	21	12,12,12
**rISC 2,8-DPTZ-DBTO$_2$**	v1	21	21,61,31
**(3 state)**	v11,v23	21	12,12,12
**ISC 2,8-DPTZ-DBTO$_2$**	v1,v11	21	3,19,19,13
**(4 state)**	v9,v23	21	3,19,19,13
	v5	21	3,11,11,11
**rISC 2,8-DPTZ-DBTO$_2$**	v1,v11	21	21,61,31
**(4 state)**	v9,v23	21	21,61,31
	v5	21	12,12,12
**ISC 3,7-DPTZ-DBTO$_2$**	v2,v9	21	12,12,12
	v22	21	12,12,12
**rISC 3,7-DPTZ-DBTO$_2$**	v2,v9	21	11,61,41
	v22	21	11,61,41

Supplementary Table 15| The CT state onset energies of 3,7-DPTZ-DBTO$_2$ in a range of solvents. N.B. These values are estimated using the dataset in Figure2b. The values used in Figure1c are more accurate and have been averaged over multiple spectra from a range of different techniques.

Solvent	CT_{ax} (eV)	CT_{eq} (eV)
MCH	3.10 ± 0.01	2.52 ± 0.01
PhMe	2.95 ± 0.01	2.42 ± 0.01
PhCl	2.89 ± 0.01	2.24 ± 0.01
MeCN	2.86 ± 0.01	N/A

Supplementary Methods

Electrochemical and spectroelectrochemical characterization (HOMO and LUMO energies, stabilization of charges)

Generation of charged species (both radical cations and radical anions) was carried out using an *in situ* electrochemical method coupled with spectroscopic techniques. From the potentials at the onset of these redox peaks it is possible to estimate the ionization potential (IP) and the electron affinity (EA), provided these potentials are expressed on the absolute potential scale i.e. with respect to the vacuum level. The absolute potential of Fc/Fc^+ in non-aqueous electrolytes is 5.1 V.[1-3] This leads to the following equations:

$$IP(eV) = |e|(E_{ox(onset)} + 5.1) \quad (1)$$

$$EA(eV) = -|e|(E_{red(onset)} + 5.1) \quad (2)$$

The HOMO-LUMO levels were determined electrochemically, using cyclic voltammetry (CV) analysis. This method estimates the electron affinity and the ionization potentials, which are similar to the HOMO and LUMO energies. [3, 4]

The electrochemical cell comprised of a working electrode, of 1 mm diameter, made from platinum, a reference electrode made from Ag/AgCl, and a platinum coil as an auxiliary electrode. Cyclic voltammetry measurements were conducted using the potentiostat Biologic SP300 at room temperature, at a potential rate of 50 mV/s, and were calibrated against a ferrocene/ferrocenium redox couple.

All solvents for the synthesis were dried before use. Other commercially available substances and reagents were used without purification. Electrochemical measurements were conducted in 1.0 mM concentrations for all compounds. Electrochemical studies were undertaken in 0.1 M solutions of Bu_4NBF_4, 99% (Sigma Aldrich) in dichloromethane (DCM) or THF solvent, CHROMASOLV®, 99.9% (Sigma Aldrich) at room temperature.

All the investigated compounds were electroactive and showed both oxidation and reduction processes (Supplementary Figure 1). 3,7-DPTZ-$DBTO_2$ had slightly lower oxidation potential ca. 0.05 eV than 2,8-DPTZ-$DBTO_2$ which corresponds to a lower ionization potential, and the same reduction potential (Supplementary Figure 1, Supplementary Table 1). The slightly lower ionization potential in 3,7-DPTZ-$DBTO_2$ corresponds to a lower HOMO energy and with a similar electron affinity energy, it means the conjugation length in 3,7-DPTZ-$DBTO_2$ is slightly higher than 2,8-DPTZ-$DBTO_2$. This is possible if the angle between donor and acceptor planes in 3,7-DPTZ-$DBTO_2$ is more deviated from its perpendicular configuration compared to compound 2,8-DPTZ-$DBTO_2$.

The HOMO energy is not the only difference between the molecules, strange behaviour is observed for 3,7-DPTZ-DBTO$_2$ during the second oxidation peak. In 3,7-DPTZ-DBTO$_2$ there are two oxidation stages, which are close to each other, meaning firstly, it is easy to miss the first one at around 0.4 V, secondly the donor groups are not electrochemically equivalent. In 2,8-DPTZ-DBTO$_2$ both donor groups (in D–A–D) are similar and they oxidize at the same potential, whereas in 3,7-DPTZ-DBTO$_2$ the donor groups are different, firstly we see oxidation of the more conjugated part of molecule and the second oxidation is from the less conjugated part of 3,7-DPTZ-DBTO$_2$. One of the donor groups deviates from a perpendicular conformation compared to the other donor group, which increases the conjugation length, whereas the second donor group remains in a similar position like in 2,8-DPTZ-DBTO$_2$. As a resulting effect during the second peak (0.54 V) the electrocrystallization occurs, which changes the CV shape and shows a strong high current de-doping peak (Supplementary Figure 1b). Oxidation of the second donor group increases the probability of π-π stacking of the molecules through first donor group resulting in aggregation and electrocrystallization of 3,7-DPTZ-DBTO$_2$ molecules. Such behaviour is not observed for 2,8-DPTZ-DBTO$_2$ and it is probably connected to the less conjugated 2,8 connections.

Solvatochromic shift observed in the fluorescence spectrum of DPTZ-DBTO$_2$ isomers.

Supplementary Figures 6 and 7a show the absorption spectra of 2,8-DPTZ-DBTO$_2$ and 3,7-DPTZ-DBTO$_2$ collected in solvents of increasing polarity. No solvatochromic shift is observed in the main $\pi\pi^*$ absorption bands of 2,8-DPTZ-DBTO$_2$ and 3,7-DPTZ-DBTO$_2$. Contrary to the absorption, the emission spectra of 2,8-DPTZ-DBTO$_2$ and 3,7-DPTZ-DBTO$_2$ are strongly affected by the solvent polarity. With increasing solvent polarity the emission further red shifts (for both molecules) and its intensity decreases (for 2,8-DPTZ-DBTO$_2$). Supplementary Figure 5a shows the 2,8-DPTZ-DBTO$_2$ absorption in a range of solvents at high concentration highlighting the bathochromic shift, which is not observed in 3,7-DPTZ-DBTO$_2$ (See Supplementary Figure 5b). The solvatochromism of the 3,7-DPTZ-DBTO$_2$ emission is shown in the main article (Figure 2b) as both the axial and equatorial CT states can be observed.

Effect of oxygen on the 2,8-DPTZ-DBTO$_2$ and 3,7-DPTZ-DBTO$_2$ steady state fluorescence

A comparison is shown between aerated (blue dashed lines) and degassed (black solid lines), steady-state fluorescence spectra of 2,8-DPTZ-DBTO$_2$ and 3,7-DPTZ-DBTO$_2$ in MCH solutions at RT (Supplementary Figure 7 and Supplementary Figure 8). Upon degassing the solution, the fluorescence integral increases by a factor of ca. 11 for 2,8-DPTZ-DBTO$_2$ and 3 for 3,7-DPTZ-DBTO$_2$. The spectral shape of the emission obtained in aerated and degassed solutions match closely.

$$\frac{I_{SS}^{dg}}{I_{SS}^{O2}} - 1 = \frac{I_{DF}}{I_{PF}} = 11 \text{ (For 2,8-DPTZ-DBTO}_2\text{)}$$

$$\frac{I_{SS}^{dg}}{I_{SS}^{O2}} - 1 = \frac{I_{DF}}{I_{PF}} = 2.6 \text{ (For 3,7-DPTZ-DBTO}_2\text{)}$$

Spectroelectrochemistry and EPR

Changes in the optical spectra upon electrochemical oxidation of the examined compounds were recorded as a function of applied potential in accordance with cyclic voltammetry

(Supplementary Figure 2). It can be observed that as the potential is sequentially increased the intensity of absorption peaks at 254 nm and 335-340 nm diminish, along with the formation of new peaks at lower energy regions of the spectra at 516-518 nm for 2,8-DPTZ-$DBTO_2$ and 3,7-DPTZ-$DBTO_2$, which could be assigned to typical phenothiazine radical cation bands,[7] which in this case are blue-shifted because of the presence of strong acceptor units in the main chain of the molecules.

The difference in position of the new band between the two molecules is relatively small (Δ=2 nm). This is probably due to strong localization of the radical cation on the phenothiazine moiety. This is supported by EPR spectroelectrochemical measurements. EPR spectra reveal hyperfine structure (Supplementary Figure 2). From the set of coupling constants obtained for both charged molecules it can be deduced that the maximum spin density of the radical cation is located on the phenothiazine core. This is also supported by the high g-factor value of 2.00549 for 2,8-DPTZ-$DBTO_2$ and 2.00565 for 3,7-DPTZ-$DBTO_2$ (Supplementary Table 2). These high values of g-factor are consistent with the localization of a large part of the spin density on the sulphur atom of the phenothiazine moiety.[8]

The spectra of electrochemically generated radical anions were measured using the same procedure (Supplementary Figure 3). Contrary to the oxidation, reduction of both compounds gives rise to no new absorption bands in the visible part of spectrum. These different spectral changes confirm that reduction and oxidation processes take place on different parts of the molecules which are strongly decoupled. This is also supported by the rather small differences in reduction potentials of 2,8-DPTZ-$DBTO_2$ and 3,7-DPTZ-$DBTO_2$, which are almost identical. It can be assumed that reduction takes place at the dibenzothiophene-*S,S*-dioxide unit. The lack of new absorption bands could be explained by the low oscillator strength of new transitions accompanying the formation of new charge carries.

The spectroelectrochemical EPR measurements (Supplementary Figure 3) confirmed that the radical anion is strongly localized on the acceptor core of the molecules. As in the case of anodic generation of charged species, in the cathodic process the radical anions exhibit multiline EPR spectra with g-value in the range of 2.00236-2.00238. The observed spectra could be simulated using hyperfine interactions of the unpaired electron with the protons present in dibenzothiophene- *S,S*-dioxide core (Supplementary Figure 4).[4] Even when the spectral shape is different, simulation of the two charged species prove the similarity of the process. In both cases, during reduction we observe two different radical anions forming signals at different g-value (Supplementary Figure 4, green, orange line). Analysis of the g-factor value gives no evidence of interactions of the unpaired electron with either the sulphur or oxygen in the acceptor core[5] (Supplementary Table 3).

Conversion from wavelength to energy scale

To ensure consistency and accurate estimates of the emission onset energy, and a truer representation of the spectral shape all emission spectra (unless explicitly stated otherwise) have been converted to an energy scale according to the Jacobian that is presented in the paper by Mooney and Kambhampati.[2]

Fitting of the exponential decays using Origin software

The exponential decay fits of the integrated time-resolved spectra were performed using the Origin fitting function ExpDec3 and a user-created ExpDec4 that had the following formats,

$$y = A_1 e^{\frac{x}{\tau_1}} + A_2 e^{\frac{x}{\tau_2}} + A_3 e^{\frac{x}{\tau_3}} + y_0,$$

and

$$y = A_1 e^{\frac{x}{\tau_1}} + A_2 e^{\frac{x}{\tau_2}} + A_3 e^{\frac{x}{\tau_3}} + A_4 e^{\frac{x}{\tau_4}} + y_0,$$

where y_0 was set to zero. As the decays are over several orders of magnitudes the statistical weighting method in Origin was used giving a weighting (w_i) to each point of,

$$w_i = \frac{1}{y_i},$$

where y_i is the function and not the dependent variable. Otherwise the higher intensity, prompt decays will contribute more greatly to the residuals and the fitting will be skewed to the shorter decays and in extreme cases the delayed fluorescence decays would not be fitted.

Conformers and Energetics

For both 2,8-DPTZ-$DBTO_2$ and 3,7-DPTZ-$DBTO_2$ there are 3 distinct conformers. These are denoted axial-axial (ax-ax), equatorial-equatorial (eq-eq) and axial-equatorial (ax-eq) and are shown below. The structures shown are the ground state energy minimised structures, which have been optimised using the DFT(PBE))[6] and a def2-SVP basis set as implemented within the Gaussian 09 quantum chemistry package[7].

When performed in the gas phase all three have equivalent energies. However, this degeneracy is split by the addition of the solvent (polarisable continuum model with the dielectric constant of dichloromethane), arising from the molecular dipole moment. This suggests that prevalence of each structure calculated here using a Boltzmann distribution (below) may be altered by the polarity of the solvent used during the synthesis.

$$\%\text{ conformer i} = \frac{\exp(-E_i/k_bT)}{\sum_j \exp(-E_j/k_bT)}$$

The HOMO-LUMO band gaps of these conformers decrease in the order of axial-axial conformers (ax-ax), an equatorial-axial conformer (eq-ax) and an equatorial equatorial conformer (eq-eq), suggesting a red shift of the lowest excited states following that order.

Charge Transfer Character

In 2,8-DPTZ-$DBTO_2$ eq-eq, the HOMO and LUMO are completely separated. The HOMO resides on the two donor groups, while the LUMO resides on the acceptor. These distributions of the frontier orbitals favour the effective formation of ICT states, as previously reported.[8] For the 3,7-DPTZ-$DBTO_2$ eq-ax, the HOMO is localized on the eq donor group, while the LUMO is delocalized on the acceptor unit.

As described in the main text, the relative shift of the ^{1}CT emission in 2,8-DPTZ-$DBTO_2$ is greater than 3,7-DPTZ-$DBTO_2$, consistent with a stronger CT character of the excited state in 2,8-DPTZ-

DBTO$_2$. This can be confirmed using the overlap of the HOMO and LUMO orbitals, calculated using DFT(M062X)[9], on the donor and acceptor involved in the lowest CT state. For 2,8-DPTZ-DBTO$_2$, the absolute orbital overlap is 0.086, while for the 3,7-DPTZ-DBTO$_2$ this is 0.140**.** The orbitals are shown in Supplementary Figure 8.

The ISC and rISC rates

As in recent previous works[1,3], insight into the ISC and rISC of these molecules requires dynamical simulations, which explicitly take into account the vibronic coupling within the triplet manifold.

The model Hamiltonians used for the D-A analogue 2,8-DPTZ-DBTO$_2$ is based upon the Linear Spin Vibronic Model Hamiltonian published in ref.[3] This Hamiltonian has the form:

Eq. 1
$$\hat{H} = \begin{pmatrix} \frac{\omega}{2}\left(\frac{\partial^2}{\partial Q_i^2} + Q_i^2\right) + E^{rel}_{^3LE} & \lambda_{Q_i} & E_{SOC} \\ \lambda_{Q_i} & \frac{\omega}{2}\left(\frac{\partial^2}{\partial Q_i^2} + Q_i^2\right) + E^{rel}_{^3CT} & E_{HFI} \\ E_{SOC} & E_{HFI} & \frac{\omega}{2}\left(\frac{\partial^2}{\partial Q_i^2} + Q_i^2\right) + E^{rel}_{^1CT} \end{pmatrix}$$

Where Q_i is the nuclear degree of freedom. In the present model, three nuclear degrees of freedom are included all of which were found, as described in ref.[3] to promote coupling between the ^3LE_D and ^3CT states. The relative energies of the three excited states (^3LE_D and $^{1,3}CT$) are shown in Supplementary Table 4. All of the other parameters used in the Hamiltonian are unchanged from ref.[3] and are shown in Supplementary Table 6.

The second Hamiltonian incorporates a higher lying ^3LE_A state (4 state model Hamiltonian for the D-A analogue 2,8-DPTZ-DBTO$_2$). This can be cast in the form below, where in this case the kinetic energy operator has been neglected from the diagonal components.

$$\hat{H} = \begin{pmatrix} \frac{\omega}{2}Q_i^2 + E^{rel}_{^3LE_D} & \lambda_{Q_i} & E_{SOC} & \lambda_{Q_i} \\ \lambda_{Q_i} & \frac{\omega}{2}Q_i^2 + E^{rel}_{^3CT} & E_{HFI} & \lambda_{Q_i} \\ E_{SOC} & E_{HFI} & \frac{\omega}{2}Q_i^2 + E^{rel}_{^1CT} & E_{SOC} \\ \lambda_{Q_i} & \lambda_{Q_i} & E_{SOC} & \frac{\omega}{2}Q_i^2 + E^{rel}_{^3LE_A} \end{pmatrix}$$

The additional parameters required for this model include the vibrational coupling between the ^3CT and ^3LE_A and the vibrational coupling between the ^3LE_A and ^3LE_D. These occur along modes 5 and 9, which correspond to acceptor torsion and flex between the D-A groups and are shown in Supplementary Table 7.

The final model Hamiltonian is a 3 state D-A model Hamiltonian of the D-A analogue 3,7-DPTZ-DBTO$_2$. This is also based upon a linear spin-vibronic Hamiltonian model and has the same form as shown in Eq. 1 above. All of the expansion coefficients of the Hamiltonian have been calculated in the same manner as for the D-A analogue 2,8-DPTZ-DBTO$_2$ and described in ref.[3] Potential energy surfaces, along the important normal modes were calculated using TDDFT(M062X)[9] within the Tamm-Dancoff approximation (TDA)[10] and a def2-TZVP basis set as implemented within the Gaussian quantum chemistry package.[7] Only modes with significant coupling between the lowest two triplet states were included into the model. This will be discussed in more detail in a forthcoming publication. All of the expansion coefficients are given in Supplementary Table 8.

The quantum dynamics were performed within a Quantics quantum dynamics package[11]. The full details the simulations for each Hamiltonian are given in Supplementary Table 14. All simulations were initiated from either the ^{1}CT state or lowest triplet state, depending on if they are related to ISC or rISC, respectively. For the rISC simulations, temperature needs to be included. Therefore, the quantum dynamics were performed within a density operator formalism of MCTDH[12]. Here the single particle functions used for the standard wavefunction implementation of MCTDH are replaced with single-particle density operators. Throughout this work we adopt a closed quantum system, meaning that no dissipative operators are included.

Synthesis: General experimental details

All reactions were carried out under an argon atmosphere unless otherwise stated. Starting materials were purchased commercially and were used as received. Solvents were dried using an Innovative Technology solvent purification system and were stored in ampoules under argon.

TLC analysis was carried out using Merck Silica gel 60 F_{254} TLC plates and spots were visualised using a TLC lamp emitting at 365, 312 or 254 nm. Silica gel column chromatography was performed using silica gel 60 purchased from Sigma Aldrich.

^{1}H and ^{13}C NMR spectroscopy was carried out on Bruker AV400. Residual solvent peaks were referenced as described in the literature[13], and all NMR data was processed in MestReNova V10.

Melting points were obtained on a Stuart SMP40 machine.

High resolution mass spectrometry was carried out on a Waters LCT Premier XE using ASAP ionisation. Samples were analysed directly as solids using N_2 at 350 °C.

Elemental analysis was performed on an Exeter Analytical E-440 machine.

3,7-dibromodibenzothiophene-*S*,*S*-dioxide was prepared as reported in the literature.[14,15]

Any stated use of hexane refers to a mixed isomers grade.

Synthesis of 3,7-Bis(phenothiazin-10-yl)dibenzothiophene-*S*,*S*-dioxide

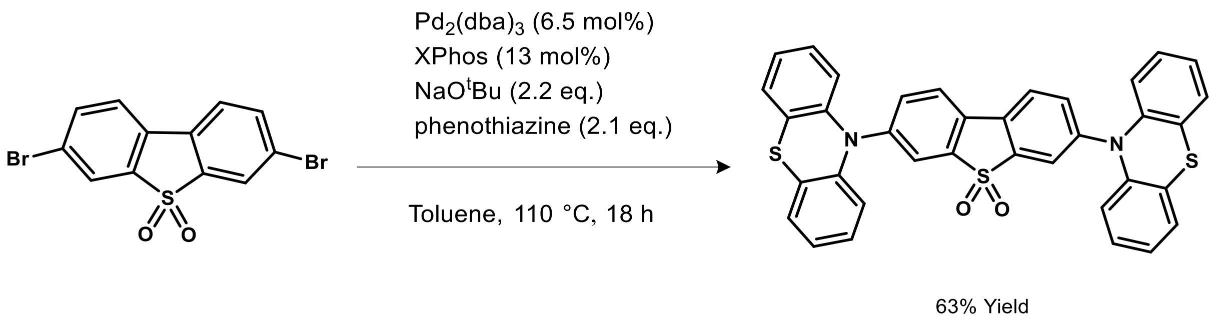

3,7-dibromodibenzothiophene-*S*,*S*-dioxide (500 mg, 1.34 mmol, 1 eq.), $Pd_2(dba)_3$ (80 mg, 87 µmol, 0.065 eq.), XPhos (83 mg, 174 µmol, 0.13 eq.) and phenothiazine (562 mg, 2.82 mmol, 2.1 eq.) were dissolved in dry toluene (20 mL) and the mixture was bubbled with argon for 30 minutes. NaO^tBu (284 mg, 2.96 mmol, 2.2 eq.) was added and reaction mixture was deoxygenated with argon for a further 15 minutes. The reaction was stirred for 18 h at 110 °C. Upon cooling, the mixture was diluted with toluene (50 mL), washed with water, and dried over Na_2SO_4, filtered and solvent removed under reduced pressure. The residue was purified by silica gel column chromatography eluting with 75:25 CH_2Cl_2:hexane (*v*/*v*). Removal of solvent gave the product as a yellow solid (518 mg, 63% yield). The title compound can be sublimed under vacuum (9×10^{-2} mbar, > 350 °C). Crystals suitable for X-ray analysis were grown over three days by layering with CH_2Cl_2: hexane.

^{1}H NMR (400 MHz, DMSO-d_6) δ 8.23 (d, *J* = 8.4 Hz, 2H), 7.77 (d, *J* = 2.1 Hz, 2H), 7.60 (dd, *J* = 8.4, 2.1 Hz, 2H), 7.35 (dd, *J* = 7.5, 1.5 Hz, 4H), 7.22 (ddd, *J* = 8.1, 7.5, 1.5 Hz, 4H), 7.11 (td, *J* = 7.5, 1.3 Hz, 4H), 6.84 (dd, *J* = 8.1, 1.3 Hz, 4H); ^{13}C NMR (100 MHz, DMSO-d_6) δ 144.5, 141.8, 139.2, 129.9, 127.83, 127.80, 127.0, 125.8, 124.9, 124.6, 121.1, 116.8; HRMS-ASAP$^+$ *m/z* calculated for $C_{36}H_{23}N_2O_2S_3$ $[M+H]^+$ 611.0922, found: 611.0925; Anal. Calc. for $C_{36}H_{22}N_2O_2S_3$ C, 70.80; H, 3.63; N, 4.59. Found: C, 70.82; H, 3.62; N, 4.56; m.p. decomp. > 350 °C.

^{1}H and ^{13}C NMR spectra

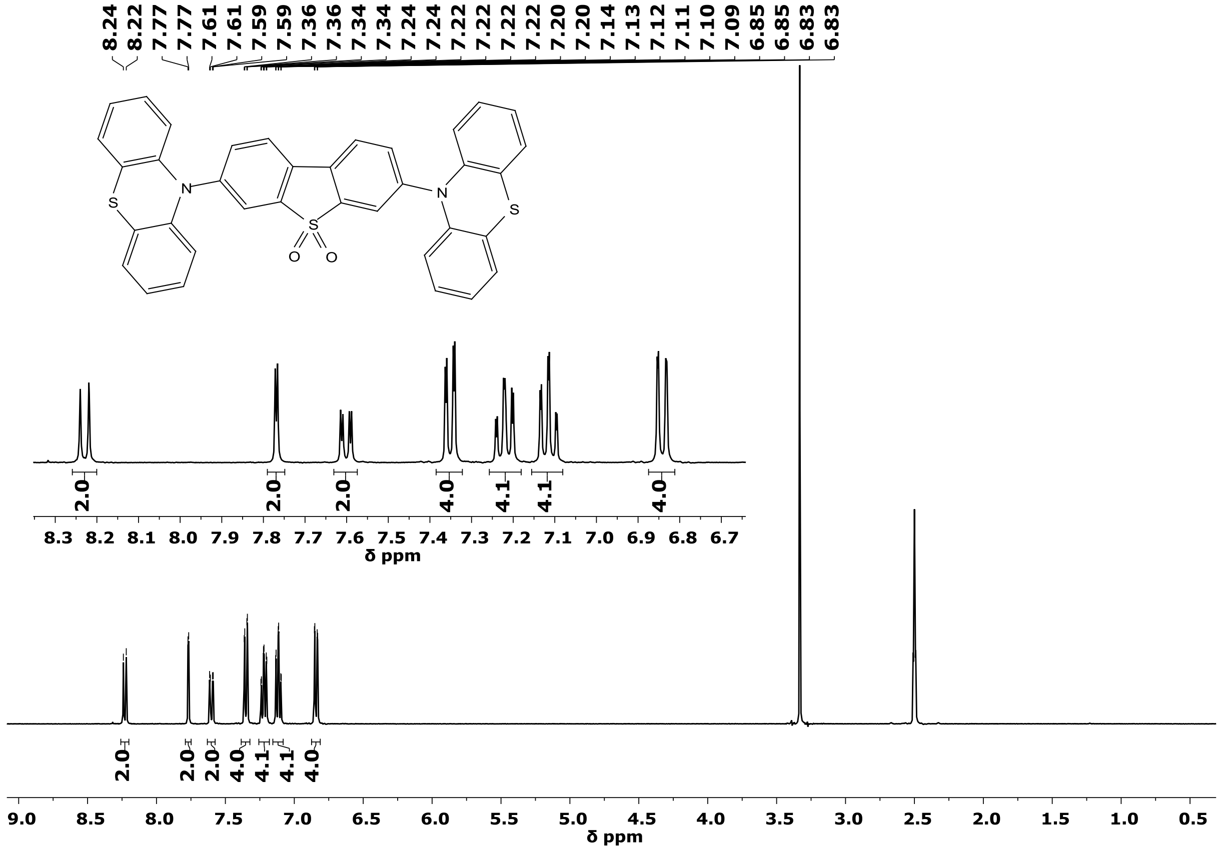

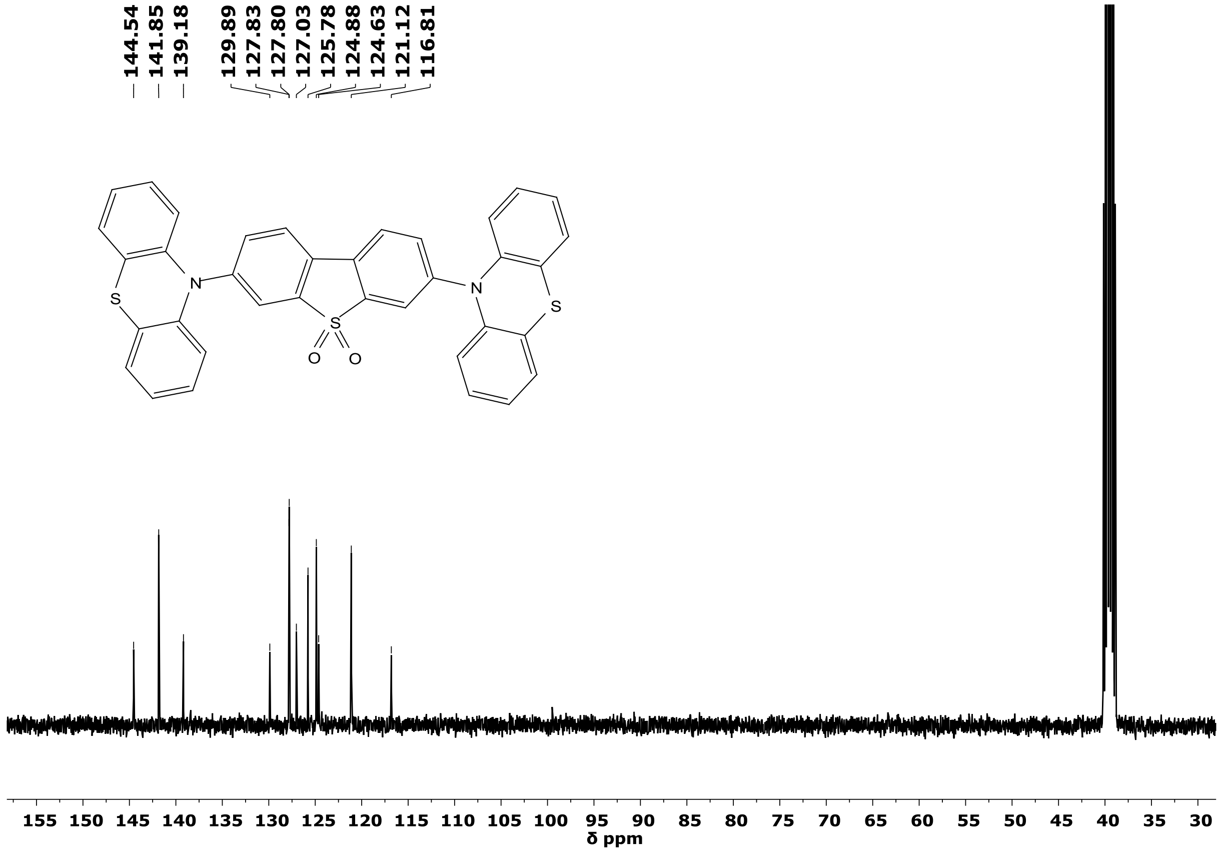

X-ray crystallography of 3,7-DPTZ-$DBTO_2$

The X-ray diffraction experiment was carried out on a Bruker 3-circle D8 Venture diffractometer with a PHOTON 100 CMOS area detector, using Cu-K_α radiation (λ=1.54184 Å) from a I µS microsource with focusing mirrors. The crystal was cooled to 120 K using a Cryostream (Oxford Cryosystems) open-flow N_2 gas cryostat. *Crystal data*: $C_{36}H_{22}N_2O_2S_3$ (M = 610.73), monoclinic, space group $P2_1/c$ (no. 14), a = 18.3663(8), b = 17.8328(8), c = 8.5964(4) Å, β = 96.749(2)°, V = 2796.0(2) $Å^3$, Z = 4, μ(CuKα) = 2.735 mm^{-1}, D_c = 1.451 g/cm^3, 20810 reflections measured (2Θ ≤ 149.2°), of which 5077 unique (R_{int} = 0.0536). Crystals showed non-merohedral twinning by a 180° rotation around the direct axis [4 0 1], i.e. the reciprocal axis **a*** (twinning law 1 0 ½ 0 -1 0 0 0 -1). The twinning pattern was deconvoluted using PLATON TwinRotMatrix program.[16] The structure was solved by direct methods using SHELXS 2013/1 software[17] and refined by full-matrix least squares using SHELXL 2014/7[18] and OLEX2[19] software, converging to R_1 = 0.053 for 4505 reflections with $I > 2\sigma(I)$ and wR_2= 0.137 on all data. Full crystallographic data has been deposited with the Cambridge Crystallographic Data Centre, dep. no. CCDC-1519688.

In the molecule of 3,7-DPTZ-$DBTO_2$, the central dibenzothiophene moiety is planar, phenothiazine substituents A and B have different orientations, with the torsion angles around the N(1)-C(3) and N(2)-C(10) bonds of 76.0° and 17.4°, respectively (Supplementary Figure 24). Consequently, the lone electron pair of N(2) is conjugated with the π-system of dibenzothiophene, while that of N(1) is instead involved in π-conjugation within the phenothiazine moiety. Thus, the N(1)-C(3) bond of 1.439(5) Å is longer than the N-C bonds within the phenothiazine moieties A (mean 1.416(5) Å), whereas the N(2)-C(10) bond of 1.406(5) Å is shorter than the N-C bonds in phenothiazine B (mean 1.438(5) Å). Both phenothiazines are folded along the N…S vectors, but A is substantially more planar than B: note the dihedral angles 153.9° (A) and 136.6° (B).

Supplementary References

1. Etherington, M. K., Gibson, J., Higginbotham, H. F., Penfold, T. J. & Monkman, A. P. Revealing the spin–vibronic coupling mechanism of thermally activated delayed fluorescence. *Nat. Commun.* **7,** 13680 (2016).

2. Mooney, J. & Kambhampati, P. Get the Basics Right: Jacobian Conversion of Wavelength and Energy Scales for Quantitative Analysis of Emission Spectra. *J. Phys. Chem. Lett.* **4,** 3316–3318 (2013).

3. Gibson, J., Monkman, A. P. & Penfold, T. J. The Importance of Vibronic Coupling for Efficient Reverse Intersystem Crossing in Thermally Activated Delayed Fluorescence Molecules. *ChemPhysChem* **17,** 2956–2961 (2016).

4. Pluczyk, S., Zassowski, P., Łapkowski, M., Schab-Balcerzak, E. & Grucela, M. Comprehensive UV-Vis and EPR spectroelectrochemical characterization of ambipolar azomethinenaphthaldiimides. *J. Electroanal. Chem.* **745,** 14–21 (2015).

5. Pluczyk, S. *et al.* UV-vis and EPR spectroelectrochemical investigations of triarylamine functionalized arylene bisimides. *RSC Adv.* **5,** 7401–7412 (2015).

6. Adamo, C. & Barone, V. Toward reliable density functional methods without adjustable parameters: The PBE0 model. *J. Chem. Phys.* **110,** 6158–6170 (1999).

7. Frisch, M. J. *et al.* Gaussian 09, Revision a 1. (2009).

8. Dias, F. B. *et al.* The Role of Local Triplet Excited States and D-A Relative Orientation in Thermally Activated Delayed Fluorescence: Photophysics and Devices. *Adv. Sci.* **3,** 1600080 (2016).

9. Zhao, Y. & Truhlar, D. G. The M06 suite of density functionals for main group

thermochemistry, thermochemical kinetics, noncovalent interactions, excited states, and transition elements: Two new functionals and systematic testing of four M06-class functionals and 12 other function. *Theor. Chem. Acc.* **120,** 215–241 (2008).

10. Hirata, S. & Head-Gordon, M. Time-dependent density functional theory within the Tamm–Dancoff approximation. *Chem. Phys. Lett.* **314,** 291–299 (1999).

11. Beck, M. The multiconfiguration time-dependent Hartree (MCTDH) method: a highly efficient algorithm for propagating wavepackets. *Phys. Rep.* **324,** 1–105 (2000).

12. Meyer, H.-D. & Worth, G. A. Quantum molecular dynamics: propagating wavepackets and density operators using the multiconfiguration time-dependent Hartree method. *Theor. Chem. Acc.* **109,** 251–267 (2003).

13. Fulmer, G. R. *et al.* NMR chemical shifts of trace impurities: Common laboratory solvents, organics, and gases in deuterated solvents relevant to the organometallic chemist. *Organometallics* **29,** 2176–2179 (2010).

14. Zhu, X. Q., Dai, Z., Yu, A., Wu, S. & Cheng, J. P. Driving forces for the mutual conversions between phenothiazines and their various reaction intermediates in acetonitrile. *J. Phys. Chem. B* **112,** 11694–11707 (2008).

15. Perepichka, I. I., Perepichka, I. F., Bryce, M. R. & Pålsson, L.-O. Dibenzothiophene-S,S-dioxide–fluorene co-oligomers. Stable, highly-efficient blue emitters with improved electron affinity. *Chem. Commun.* 3397 (2005).

16. Spek, A. L. Single-crystal structure validation with the program PLATON. *J. Appl. Crystallogr.* **36,** 7–13 (2003).

17. Sheldrick, G. M. A short history of SHELX. *Acta Crystallogr. Sect. A Found. Crystallogr.* **64,** 112–122 (2007).

18. Sheldrick, G. M. Crystal structure refinement with SHELXL. *Acta Crystallogr. Sect. C Struct. Chem.* **71,** 3–8 (2015).

19. Dolomanov, O. V., Bourhis, L. J., Gildea, R. J., Howard, J. A. K. & Puschmann, H. OLEX2: A complete structure solution, refinement and analysis program. *J. Appl. Crystallogr.* **42,** 339–341 (2009).
